# Design and testing of a soft parallel robot based on pneumatic artificial muscles for wrist rehabilitation

**DOI:** 10.1038/s41598-020-80411-0

**Published:** 2021-01-14

**Authors:** Yaxi Wang, Qingsong Xu

**Affiliations:** 1 Department of Electromechanical Engineering, Faculty of Science and Technology, University of Macau, Taipa, Macau China; 2 Zhuhai UM Science & Technology Research Institute, Zhuhai, Guangdong China

**Keywords:** Electrical and electronic engineering, Mechanical engineering, Rehabilitation

## Abstract

Wrist rehabilitation is needed to help post-stroke and post-surgery patients recover from wrist fracture or injury. Traditional rehabilitation training is conducted by a therapist in a hospital, which hinders timely treatment due to the corresponding time and space constraints. This paper presents the design and implementation of a soft parallel robot for automated wrist rehabilitation. The presented wrist rehabilitation robot integrates the advantages of both soft robot and parallel robot structures. Unlike traditional rigid-body based rehabilitation robots, this soft parallel robot exhibits a compact structure, which is highly secure, adaptable, and flexible and thus a low-cost solution for personalized treatment. The proposed soft wrist-rehabilitation robot is driven by six evenly distributed linear actuators using pneumatic artificial muscles and one central linear electric motor. The introduced parallel-kinematic mechanism design enables the enhancement of the output stiffness of the soft robot for practical use. An electromyography sensor is adopted to provide feedback signals for evaluating the rehabilitation training process. A kinematic model of the designed robot is derived, and a prototype is fabricated for experimental testing. The results demonstrate that the developed soft rehabilitation robot can assist the wrist to realize all the required training motions, including abduction-adduction, flexion-extension, and supination-pronation. The compact and lightweight structure of this novel robot makes it convenient to use, and suitable rehabilitation training modes can be chosen for tailored rehabilitation at home or in a hospital.

## Introduction

Stroke has become the main cause of abnormal human death. According to the global burden of disease research, more than 10 million strokes occur every year worldwide, and the number of people disabled by stroke is up to 116 million^1^. Stroke will induce damage to the central nervous system. One of the injuries is loss of wrist motion. The wrist is an important joint that connects the hand and arm. Abnormal and frequent use of the wrist could also induce a wrist fracture or injury. Thus, rehabilitation training is essential for patients with stroke and wrist injuries, with the goal of helping patients recover to normal status^2^. The traditional rehabilitation training method is based on one-on-one training between a therapist and a patient. The therapist helps the patient perform repeated training; however, this approach has many inconveniences, and the cost is high^3^. For instance, during the COVID-19 epidemic, face-to-face treatment in hospitals has been suspended. Alternatively, wrist rehabilitation robots provide a solution to assist patients in rehabilitation training. Such robots make it possible for patients to freely enjoy rehabilitation treatment at home, which can reduce the workload of therapists and expenditure of the patients. Generally, wrist rehabilitation robots are characterized by safety, flexibility, efficiency, and practicality^4–6^.

In previous work, several wrist rehabilitation robots have been proposed. For example, Oblak et al. designed a rehabilitation robot for the motion training of wrists and arms^7^. This full-featured upper limb rehabilitation robot can perform rehabilitation training in arm mode and wrist mode with its a haptic drive. Krebs et al. designed a robot called MIT-MANUS, which assists patients in quantifying exercise and allows patients to complete interactive video games for realizing rehabilitation^8^. However, this robot structure has a large footprint size, high cost, and complicated operation. Moreover, this robot is suitable for use only in hospitals; it is not appropriate for patients to perform rehabilitation training independently with this robot. Later, Squeri et al. designed a progressive wrist rehabilitation robot that uses an adaptive control method to achieve wrist rehabilitation by vibration. It achieves three-degrees-of-freedom (3-DOF) motion of the wrist, including extension, pronation, and flexion^9^. However, such a rehabilitation robot must be worn by the patient, and it has a rigid structure, which exhibits a certain degree of risk. Aabdallah et al. designed a compact wrist rehabilitation device for achieving two kinds of motion of the wrist, which detects muscle activity by using electromyography (EMG) sensors^10^. However, limited by its low mobility, the robot cannot drive the wrist to achieve most exercises and requires the patient’s self-motion. Similarly, Allington et al. designed a wrist rehabilitation robot with only two DOF^11^. The cylinder drive needs to account for the nonlinear effects caused by static friction and must reduce the effects of friction. Lan et al. designed an upper limb exoskeleton robot using series elastic actuators (SEAs)^12^. It adopts spherical and crank slider mechanisms, which have a light weight and can be precisely controlled. However, the rigid-body exoskeleton structure has the disadvantage of being unsafe and may cause secondary injury to the patient to a certain extent. Abhishek et al. designed a 3-Revolute Prismatic Spherical (RPS) RiceWrist parallel rehabilitation robot that can achieve kinaesthetic feedback during rehabilitation. It has a compact structure and low friction and can achieve 4-DOF movement. Due to the rigid structure of the exoskeleton, there is also a certain degree of insecurity^13^. In addition, Krebs et al. designed another wrist rehabilitation robot^14^. However, it cannot fit the actual movement tracking of the wrist, and the rehabilitation effect is not satisfactory. Moreover, Spencer et al. developed a 3-DOF wrist rehabilitation robot^15^, which is prone to lock-up, but the motion control is not precise enough. Additionally, several 5-DOF exoskeleton rehabilitation robots have been developed in the literature^16^. For patients with wrist dysfunction, a 3-DOF exoskeleton, i.e., a portable wrist rehabilitation robot, was designed in the literature^17^. However, such devices have relatively large footprints.

To overcome the shortcomings of previous designs, including complex structures, safety risks, complicated operations, and limited-DOF motions, a new wrist rehabilitation robot for practical use is needed. Specifically, soft robots provide a solution^18^. Unlike traditional rigid-body robots, soft robots deliver motion or force with soft materials, resulting in safer interactions with humans^19^. Nevertheless, soft robots usually exhibit a large compliance with relatively low stiffness^20,21^. It is challenging to devise a soft robot with sufficiently large stiffness for use in wrist rehabilitation. To this end, a new soft parallel robot is designed in this work by integrating the concepts of soft robots and parallel robots. Unlike the serial-kinematic robot with multiple links and joints connected in series (such as a robotic arm), the end-effector of a parallel robot is connected to the base by multiple limbs in parallel, providing a larger output stiffness^22,23^. Hence, the proposed soft parallel robot exhibits the advantages of both soft robots and parallel robots for wrist rehabilitation. To the best of our knowledge, this is the first of its kind presented in the literature.

In particular, the soft parallel wrist rehabilitation robot presented in this work was designed based on pneumatic artificial muscles (PAMs). PAM is an emerging soft actuator that has been used in soft robotics^24,25^. As a type of soft-body actuator, a PAM requires only air pressure to produce its movement^26^. The control and operation of PAMs are easy to realize, and the structure is compact and very safe. The main structure of the wrist rehabilitation robot employs the 6-SPS/PS parallel mechanism (S means spherical joint and P means prismatic joint)^27^. Compared with the serial kinematic mechanism, the parallel mechanism exhibits a more compact structure, lower inertia, and higher precision. Unlike traditional rigid-body wrist rehabilitation robots, the designed soft rehabilitation robot provides a safe interface, which increases the motion range of rehabilitation movement. Moreover, it can adjust the training range of the bending angle, which enables higher flexibility and safety.

## Design of the soft wrist rehabilitation robot

**Figure 1:**
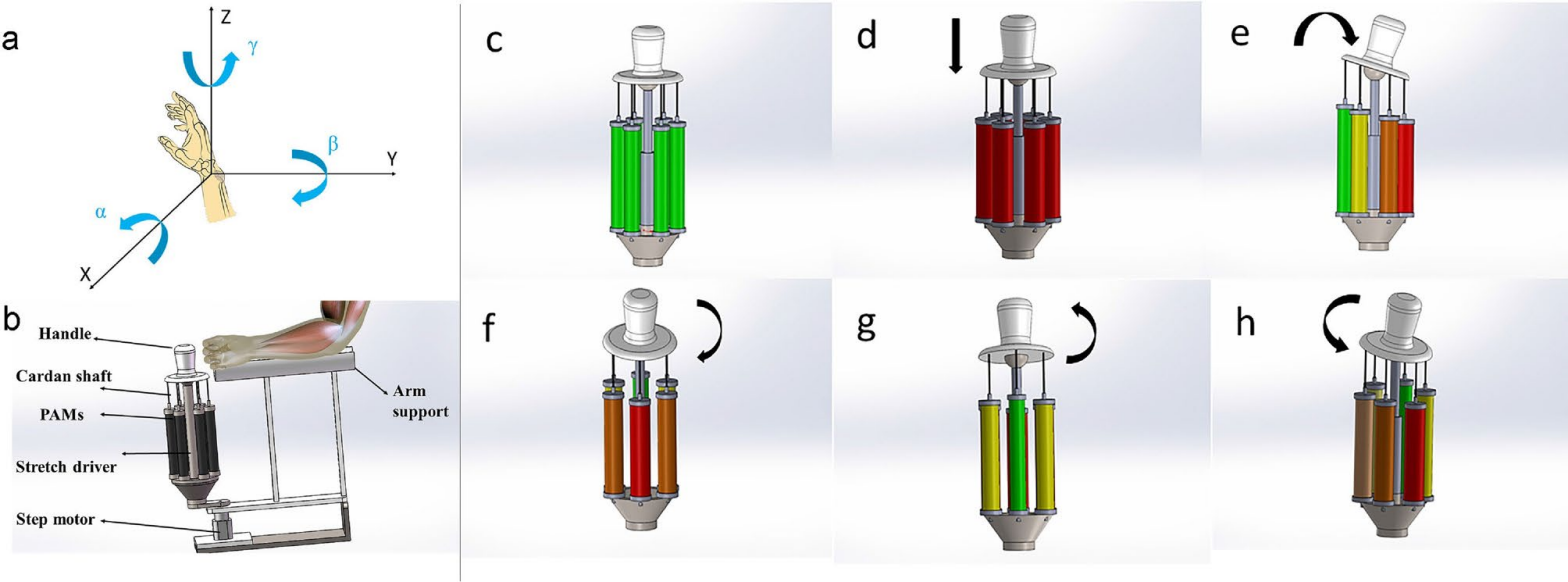
Illustration of the required motions for wrist rehabilitation. (**a**) Three rotational motions of the wrist; (**b**) CAD model of the soft parallel robot, (**c**–**h**) demonstration of the expected rehabilitation motion. (**c**) Initial state; (**d**) compressed state; (**e**) supination state; (**f**) abduction state; (**g**) adduction state; (**h**) pronation state.

According to different mechanical structures, wrist rehabilitation robots can be divided into terminal-driven rehabilitation robots and exoskeleton rehabilitation robots. This work focuses on terminal-driven rehabilitation robots owing to their better flexibility. As shown in Fig. 1a, according to the movement of the wrist joint, the spatial motion can be simplified into 3-DOF rotational motion. Specifically, the movement of the wrist includes three typical movements: abduction–adduction, flexion–extension, and supination-pronation motions^28,29^.

To describe the movement of the wrist, the three rotational movements are represented by three-dimensional coordinates in space (see Fig. 1a). The central axis of the forearm is along the Z-axis. The flexion–extension of the wrist joint and its bending lie in the ZOY plane around the X-axis. The abduction and adduction of the wrist corresponds to rotation around the Y-axis in the ZOX plane. The supination-pronation position of the wrist joint is moved around the Z-axis in the XOY plane. In addition, the corresponding angles of rotation are expressed by the flexion–extension angle α, abduction–adduction angle β , and supination-pronation angle γ. These three angles have normal ranges of − 73°–71°, − 19°–33°, and − 71°–86°, respectively^30^.

The purpose of the wrist rehabilitation robot is to drive the human wrist to perform the three major motions to help the patient recover to normal health status.

### Operation mechanism of the soft rehabilitation robot

Figure 1b shows a computer-aided design (CAD) model of the proposed soft parallel wrist rehabilitation robot. The structure of the soft wrist rehabilitation robot is designed based on a parallel mechanism with six limbs. Each of the six limbs is fabricated by a PAM. The stretch drive in Fig. 1b refers to the linear motor, which is used to cooperate with PAMs to move and support the moving platform and ultimately realize all the rehabilitation training actions that drive the wrist. In view of the required motions for wrist rehabilitation, the designed parallel mechanism provides redundant actuation. That is, the adoption of six PAMs provides a redundancy for the drive, which eliminates the singularity problem and offers a more reliable actuation.

Considering the elasticity of the PAM and steel wire, the structure can be categorized as a 6-SPS/PS parallel mechanism. The central PS limb is driven by an electric cylinder, which is used to constrain unnecessary movement, such as external swing movement. The upper end of the PS limb is a spherical joint, which can support the handle to realize motion in various directions. The advantage of this design is that it is more stable, along with better limb support and stability. To express more clearly the expected working state of the wrist rehabilitation robot, the expected rehabilitation motions are illustrated in Fig. 1c–h, including the initial compression state and the abduction–adduction, flexion–extension, and supination-pronation motions. When the applied air pressure causes the PAMs to drive the platform to swing in the left and right directions, abduction–adduction rehabilitation action can be realized, and when the stepper motor rotates, flexion–extension rehabilitation training action can be realized. When the applied air pressure causes the PAMs to drive the moving platform to swing in the front and rear directions, supination-pronation rehabilitation can be achieved.

**Figure 2:**
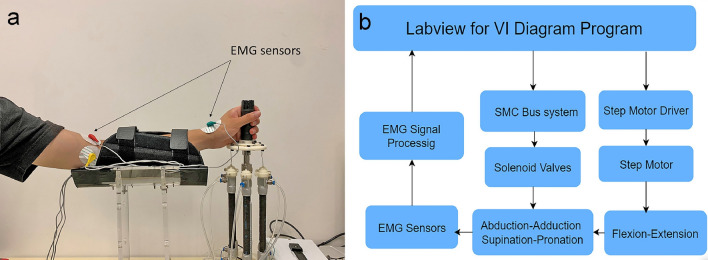
Experimental setup of the wrist rehabilitation robot. (**a**) Photograph of the fabricated prototype of the wrist rehabilitation robot; (**b**) control hardware connection diagram of the system realized by LabVIEW software.

The developed prototype is shown in Fig. 2a, and the hardware connection scheme is given in Fig. 2b. Before starting the rehabilitation work, the upper limb is placed on the arm support, and the handle is held by the palm. The PAMs and electric cylinder are simultaneously controlled (for contraction) to reach the initial state. Then, the specific aerodynamic muscle pressure and releasing air pressure can be governed to achieve the abduction–adduction motion of the wrist. The flexion–extension action is realized by the stepper motor, which is mounted at the bottom of this structure. The stepper motor rotates to drive the wrist to achieve the bending action. Finally, by applying and releasing the air pressure to all the PAM cycles in a particular order, the wrist can be driven to achieve the supination-pronation action.

## Kinematic modelling and finite element analysis (FEA) simulation of the rehabilitation robot

**Figure 3:**
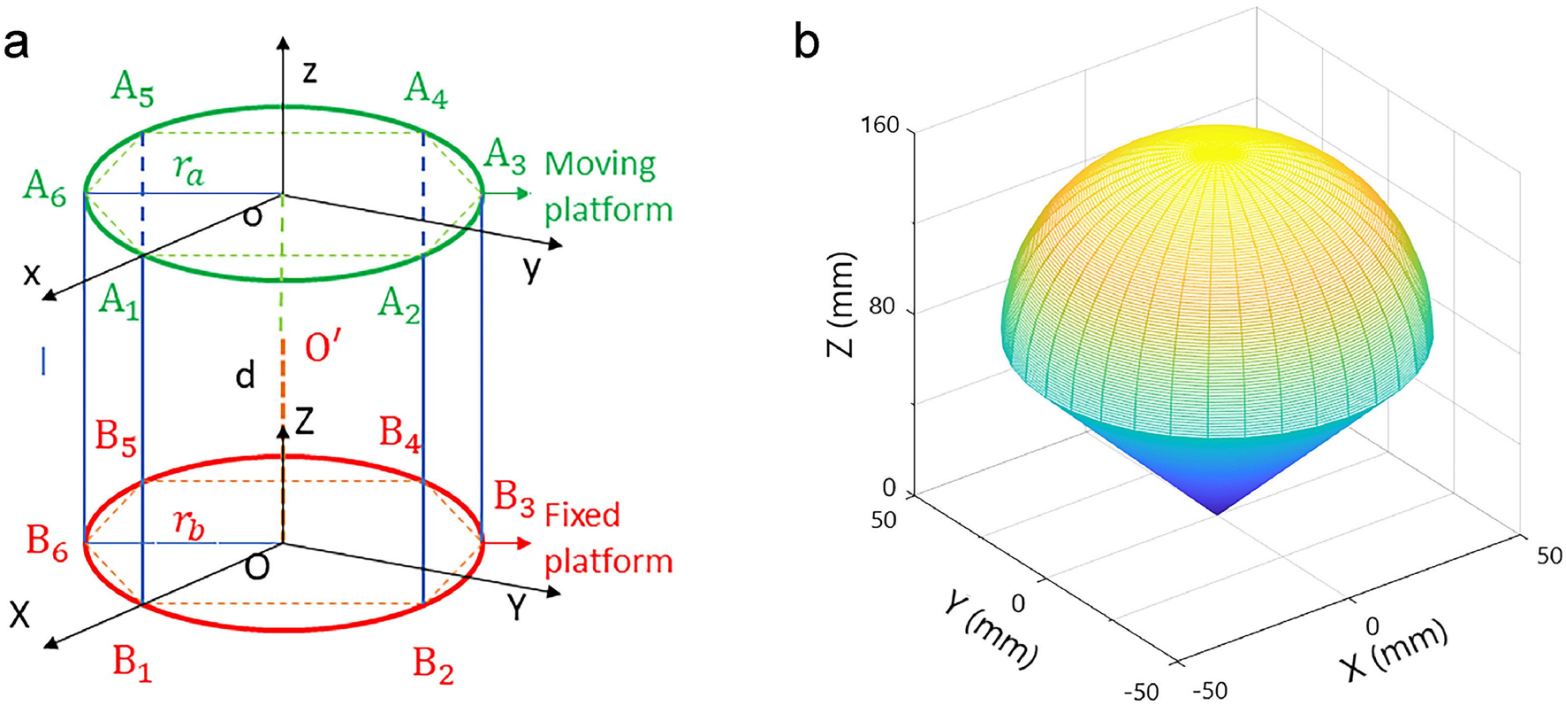
Kinematics and workspace analysis of the parallel robot. (**a**) The assigned reference coordinate systems; (**b**) spatial workspace of the moving platform of the wrist rehabilitation robot obtained by MATLAB software.

To facilitate the kinematic analysis, the coordinate systems of the moving platform (o-xyz) and fixed platform (O-XYZ) are established, as shown in Fig. 3a. The six vertical lines (in blue) represent the six PAM actuators^19^. The moving platform is connected to the six actuators by elastic steel wires. The hinge point *A*_*i*_ (*i* = 1 to 6) on the moving platform and hinge point *B*_*i*_ (*i* = 1 to 6) on the fixed platform are located in the corresponding coordinate planes. In addition, *d* represents the length of the electric cylinder. The circumscribed circle radii of the moving and fixed platforms are *r*_*a*_ and *r*_*b*_, respectively. Based on the above settings, the kinematics and workspace of the designed 6-SPS/PS parallel structure are analysed below.

### Kinematic modelling

Kinematic modelling involves both inverse and forward kinematic analyses. The purpose of inverse kinematic modelling is to solve the length *l*_*i*_ (*i* = 1 to 6) of the six branches and length *d* of the central electric cylinder for the required rehabilitation training action. In contrast, given the length of the seven branches, the objective of the forward kinematics is to calculate the pose of the moving output platform^31^.

Concerning a parallel mechanism, the inverse kinematics problem is straightforward to solve based on geometric analysis. In contrast, the forward kinematics problem is more complicated to solve^32–34^. Although the analytical method can find all the possible solutions of the pose for the output platform, the solving processes are rather complicated for practical applications. Hence, the forward kinematics of the 6-SPS/PS parallel mechanism are solved by resorting to the numerical iterative method^35^.

### Inverse and forward kinematic analyses

Concerning the 6-SPS/PS soft wrist rehabilitation parallel robot, the purpose of inverse kinematic analysis is to solve the length *l*_*i*_ (*i* = 1 to 6) of the six branches and length *d* of the central electric cylinder for the given rehabilitation training action. Referring to the geometric relationship and initial pose values (see Fig. 3a), the length of the *i*th branch can be obtained as *l*_*i*_ =|$\overrightarrow{B_{i}A_{i}}$| (for *i* = 1 to 6) and *d* =|$\overrightarrow{Oo}$|.

In addition, the objective of the forward kinematic analysis is to calculate the pose of the moving platform given the length of the seven branches. Usually, solving a series of nonlinear equations with an analytical method or a numerical method is a complex problem. Theoretically, the analytical method requires a large number of matrix operations. Although the analytical method can find all the possible solutions of the pose, the solving processes are extraordinarily complicated for practical applications. As the inverse kinematics of the 6-SPS/PS parallel mechanism have been derived easily, the forward kinematics of the 6-SPS/PS parallel mechanism are solved by an iterative method based on the inverse kinematic solution, which is explained as follows.

First, it is assumed that the initial pose is (α_0_, β_0_, γ_0_,  *x*_0_, *y*_0_, *z*_0_). This initial pose is very different from the desired pose. According to the inverse kinematic solution, the assumed initial length of the branch ${l}_{i}^{1}$ can be obtained. Then, if the length *l*_*i*_ of each branch is known, the difference between the length of the *i*th branch at the assumed initial pose and the corresponding length with actual input is recorded by δ *l*_*i*_. That is,

$$ \delta l_{i} = l_{i} - l_{i}^{1} $$

The length of the branch depends on the pose and can be described with the following function.

$$ l_{i} = l_{i} (\alpha ,\beta ,\gamma ,x,y,z) $$

where *i* = 1 to 6. The same procedure applies to the length *d* of the central electric cylinder.

The change in the pose of the moving platform is expressed by (δα_1_, δβ_1_, δγ_1_, δ*x*_1_, δ*y*_1_, δ*z*_1_), which can be calculated by

3 $$\left[\begin{array}{c}\mathrm{\delta d}\\\updelta {l}_{1}\\\updelta {l}_{2}\\\updelta {l}_{3}\\\updelta {l}_{4}\\\updelta {l}_{5}\\\updelta {l}_{6}\end{array}\right]=\left[\begin{array}{cccccc}\frac{\partial d}{\partial \alpha }& \frac{\partial d}{\partial \beta }& \frac{\partial d}{\partial \gamma }& \frac{\partial d}{\partial x}& \frac{\partial d}{\partial y}& \frac{\partial d}{\partial z}\\ \frac{\partial {l}_{1}}{\partial \alpha }& \frac{\partial {l}_{1}}{\partial \beta }& \frac{\partial {l}_{1}}{\partial \gamma }& \frac{\partial {l}_{1}}{\partial x}& \frac{\partial {l}_{1}}{\partial y}& \frac{\partial {l}_{1}}{\partial z}\\ \frac{\partial {l}_{2}}{\partial \alpha }& \frac{\partial {l}_{2}}{\partial \beta }& \frac{\partial {l}_{2}}{\partial \gamma }& \frac{\partial {l}_{2}}{\partial x}& \frac{\partial {l}_{2}}{\partial y}& \frac{\partial {l}_{2}}{\partial z}\\ \cdot & \cdot & \cdot & \cdot & \cdot & \cdot \\ \cdot & \cdot & \cdot & \cdot & \cdot & \cdot \\ \frac{\partial {l}_{6}}{\partial \alpha }& \frac{\partial {l}_{6}}{\partial \beta }& \frac{\partial {l}_{6}}{\partial \gamma }& \frac{\partial {l}_{6}}{\partial x}& \frac{\partial {l}_{6}}{\partial y}& \frac{\partial {l}_{6}}{\partial z}\end{array}\right]\left[\begin{array}{c}\begin{array}{c}\updelta {\alpha }_{1}\\\updelta {\beta }_{1}\\\updelta {\gamma }_{1}\end{array}\\ \begin{array}{c}\updelta {x}_{1}\\\updelta {y}_{1}\\\updelta {z}_{1}\end{array}\end{array}\right]$$

The first step of pose variation (δα_1_, δβ_1_, δγ_1_, δ*x*_1_, δ*y*_1_, δ*z*_1_) can be obtained by δ*l*_*i*_, and the pose value can be updated after the first correction:

$$ x_{{1}} = x_{0} + \delta x_{{1}} $$

$$ y_{{1}} = y_{0} + \delta y_{{1}} $$

6 $$ z_{{1}} = z_{0} + \delta z_{{1}} $$

7 $$ \alpha_{{1}} = \alpha_{0} + \delta \alpha_{{1}} $$

8 $$ \beta_{{1}} = \beta_{0} + \delta \beta_{{1}} $$

9 $$ \gamma_{{1}} = \gamma_{0} + \delta \gamma_{{1}} $$

Then, according to the updated pose (α_1_, β_1_, γ_1_, *x*_1_, *y*_1_, *z*_1_) and inverse kinematic solution, a new branch length *l*_*i*2_ can be obtained. The new value of branch length will gradually approach the input length of the branch. In the same way, after the second correction, an updated branch length is obtained and compared with the input value. The above iterative process is then repeated.

During the iterative process, when max |δ*H*_*in*_| is smaller than the allowable error η, the iterative process can be stopped, and max |δ*H*_*in*_| represents the obtained maximum deviation. The resulting pose (α_*n*_, β_*n*_, γ_*n*_, *x*_*n*_, *y*_*n*_, *z*_*n*_) is the desired forward kinematic solution.

### Workspace evaluation

The workspace of the 6-SPS/PS parallel wrist rehabilitation robot is a collection of working points that can be reached by the reference point of the moving platform. The size of the workspace determines the range of motion of the terminal handle, which is the motion range of the wrist. Unlike the serial-kinematic robot, the workspace of the 6-SPS/PS parallel rehabilitation robot is more complicated. It is influenced by many parameters, such as the diameters of the moving platform and the fixed platform and the variation range of the branches.

Since the physics and structure of the parallel mechanism constrain the pose of the mechanism, the range of branch lengths should be considered for obtaining the workspace of the end-effector (moving platform)^36^. In particular, the contraction range of each branch meets the following condition:

$$ l_{{{\text{min}}}} \le l \le l_{{{\text{max}}}} $$

where *l*_max_ and *l*_min_ represent the maximum and minimum lengths of the branch, respectively. In addition, the length variation of the electric cylinder and the rotation angle of the branch should meet the following constraints:

$$ d_{{{\text{min}}}} \le d \le d_{{{\text{max}}}} $$

$$ 0 \le \varphi \le \varphi_{{{\text{max}}}} $$

where *d*_max_ and *d*_min_ denote the maximum and minimum lengths of the electric cylinder, respectively, and φ_max_ represents the maximum angle of the branches.

After a series of polar radius and polar angle search calculations, the volume of the sub-workspace (*V*) can be calculated by taking into consideration the resulting discrete points:

$$ V = \frac{1}{2}\sum\nolimits_{i} {\left( {\delta \theta \rho_{i}^{2} } \right)} $$

where δ_θ_ is the torsion angle and ρ_*i*_ is the polar radius value of the *i*th boundary calculation. According to the inverse kinematic solution and the constraints of the workspace, the numerical method with boundary values is used to generate the workspace of the 6-SPS/PS parallel rehabilitation robot. The workspace consists of a myriad of discrete points. If the corresponding inverse kinematic solution satisfies the constraint conditions, the discrete point is considered to be located in the workspace. With the given kinematic parameters of the 6-SPS/PS parallel wrist rehabilitation robot, the spatial workspace is simulated using MATLAB software based on the boundary numerical method. The result is shown in Fig. 3b. It is a collection of discrete points that can be reached at a specific reference point (i.e., the centre of the handle) on the moving platform.

### Modelling and evaluation of PAM behaviour

A PAM is a tensile actuator that mimics the natural movement of a muscle. It consists of contractible tubing and appropriate connectors. The contractible tubing is composed of a rubber diaphragm with a non-crimped fibre made of aramid yarns inside. The diaphragm provides a hermetic seal enclosing the operating medium^37^. The yarns serve as a reinforcement for transmitting the power. When an internal pressure is applied, the diaphragm extends in the circumferential direction. This creates a tensile force and a contraction motion in the longitudinal direction. The usable tensile force is maximal at the start of the contraction and then reduces with increasing pressure^38,39^.

In particular, the air pressure (*p*) in the internal chamber governs the length of the PAM and determines the external load^40^. The length change is defined as *w*, the PAM actuator volume is *v*, and the load force is *T*. Under static conditions, the PAM actuator’s static load characteristics can be derived as

14 $$T=p\frac{dv}{dw}$$

*H* is defined as the compliance of the PAM actuator. The compliance is calculated by the reciprocal of stiffness, which can be expressed as follows.

15 $$H=\frac{1}{K}=-\frac{d{v}^{2}}{d{w}^{2}}-N\frac{p+{p}_{0}}{v}{\left(\frac{dv}{dw}\right)}^{2}$$

where *N* is the polytropic exponent and *p*_0_ represents the ambient pressure (kPa).

### Simulation results

**Figure 4:**
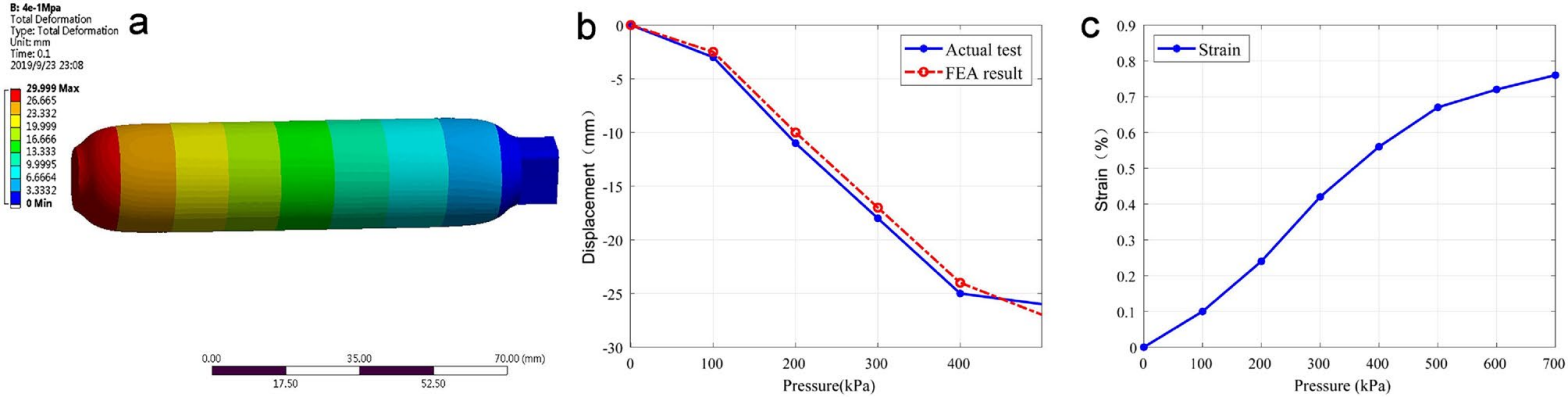
Performance evaluation results of the PAM actuator. (**a**) Finite element simulation results of total deformation of PAM obtained by ANSYS software; (**b**) total displacement comparison of FEA simulation and experimental test results; (**c**) total equivalent strain versus the applied pressure.

To reveal the feasibility and efficiency of PAM materials functioning as actuators in soft wrist rehabilitation robots, a simulation study with FEA was performed by considering their material properties^41^. Specifically, a 3-D model of the actuator is built in ANSYS software; additionally, the properties of the rubber material are defined, and the thin layer of the reticular fibre outside the model is restricted. As the working principle of the PAM is based on the Mc-Kibben muscle, a simulation study is performed with a one-end fixed, one-end free condition by applying different pressure values (0–400 kPa) at the input end. For instance, the simulation result of total deformation is shown in Fig. 4a, which illustrates the total displacement with an input pressure value of 400 kPa.

According to the total deformation displacement, it is observed that the PAM actuator undergoes an expansion of the thin air pressure, an increase in diameter, a shortening of the axial length, and a gradual shortening with an increase in the pressure value. Based on the simulation results, the relationship between the PAM displacement and air pressure value and the relationship between the PAM strain and air pressure value are obtained, as shown in Fig. 4b and c, respectively.

To further evaluate the performance of the PAM for driving wrist movement, experimental testing of the PAM was conducted by applying pressure values of 0–500 kPa. Experimental results are also shown in Fig. 4b. The maximum difference between the FEA and experimental results is below 1.0 mm.

As depicted in Fig. 4b, there is a nonlinear relationship between the PAM displacement and air pressure value. According to the PAM pressure-contraction relationship, a fitting curve is generated as follows.

$$ q = 0.013p^{2} + 0.41p - 97.5 $$

where *q* is the percentage of contraction. The pressure-contraction relationship indicates the quantitative correspondence between the pressure value and PAM contraction, which provides fundamental information for controlling the wrist rehabilitation robot in experimental implementation. Thus, the degree of contraction of the PAM actuator can be adjusted by applying different pressures, which adjust the wrist motion according to the forward kinematic solution given in Eqs. (4)–(9).

## Testing results of wrist rehabilitation application

**Figure 5:**
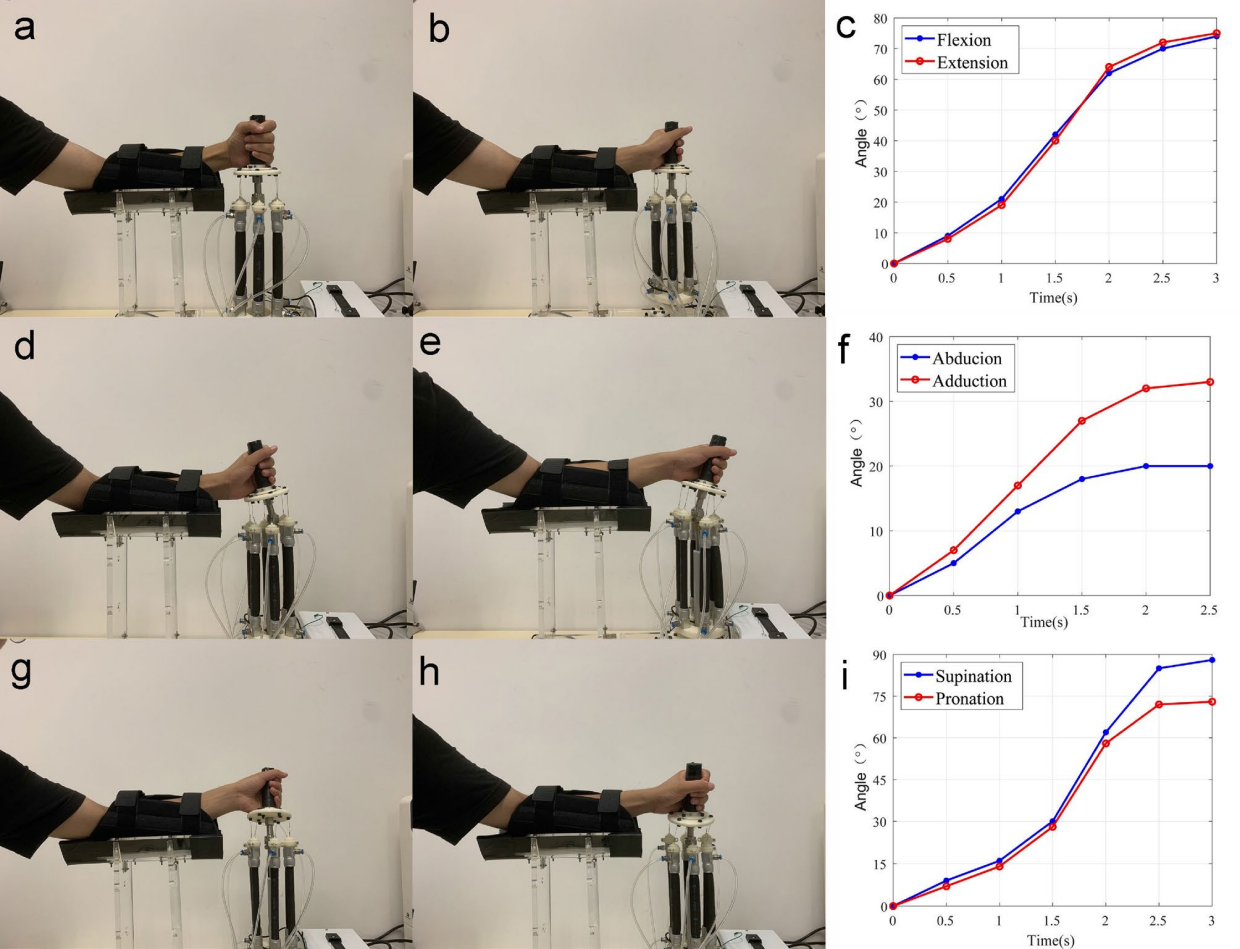
Experimental testing results of wrist rehabilitation with different training modes. (**a**,**b**) flexion–extension motion, (**c**) angle of flexion–extension rehabilitation training state; (**d**,**e**) abduction–adduction motion, (**f**) angle of abduction–adduction rehabilitation training state; (**g**,**h**) supination-pronation motion, (**i**) angle of supination-pronation rehabilitation training state.

Based on the developed soft parallel robot, a wrist rehabilitation test was performed. A set of programs was written in LabVIEW software to realize different training motions, including abduction–adduction, flexion–extension, and supination-pronation, representing all of the required movements of the wrist. In addition, the length of the PAM actuator was controlled by applying different analogue voltage signals. Thus, several sets of action procedures can be assigned, and the patient can choose different rehabilitation modes according to the actual degree of injury. Snapshots of the experimental studies are illustrated in Fig. 5, which shows three states of the rehabilitation training modes. For demonstration purposes, the experiments (see Figs. 2 and 5) were conducted by an author of this work, and informed consent was obtained.

In addition, to verify the poses obtained by kinematic analysis, the angle changes under three kinds of rehabilitation training modes were tested experimentally. The results are shown in Fig. 5c,f,i. Regarding the angle measurement in Fig. 5c,f,i, an image-based angle measurement tool was adopted. During the rehabilitation training, a camera was used to record the motion images in real time. Then, a fixed time node was selected, and the image was processed. The angle between the moving platform and the plane can be determined by measuring the target angle. For instance, in the flexion state, the angle changes from 0° to 75° within the motion range, while the expected angle is 73°, corresponding to an error of 2.7%. This error is mainly induced by the nonlinearity of the PAM actuator material and the error in the applied air pressure value. This error value is negligible because the effect of this error on the final rehabilitation training effect is very small. As mentioned earlier, according to ergonomics, the ranges of motion angles for the 3-DOF motion of the wrist are − 19°–33° for abduction–adduction, − 73°–71° for flexion–extension, and − 71°–86° for supination-pronation. By selecting these angle ranges as the predetermined intervals, the practical angle ranges are obtained as tabulated in Table 1.

**Table 1:** Comparison of the desired and achieved motion ranges.

Number	Action	Required	Actual
(a)	Abduction	0°–19°	0°–19°
	Adduction	0°–33°	0°–34°
(b)	Flexion	0°–73°	0°–75°
	Extension	0°–71°	0°–73°
(c)	Supination	0°–86°	0°–88°
	Pronation	0°–71°	0°–73°

On the basis of the actual measurement and comparison, it can be concluded that the handle can drive the wrist to reach the desired position. Furthermore, the maximum error is only 3.3%, which is within the design tolerance (5%). This allows extreme rehabilitation training to be performed with the designed wrist rehabilitation robot. The achievement of multiple rehabilitation modes enhances the versatility of the rehabilitation robot.

### Training process evaluation

To evaluate the training process, the time-domain analysis of the myoelectric signals is conducted by using the average absolute value (AAV) and variance (VAR) of the eigenvalues of the EMG signal. They are calculated below.

17 $$AAV=\frac{1}{N}{\sum }_{i}\left|{X}_{i}\right|$$

18 $$VAR=\frac{1}{N-1}{\sum }_{i}\left({X}_{i}^{2}\right)$$

where *N* is the number of sampling points in the concerned time period and *X*_*i*_ is the amplitude of the EMG signal at the *i*th sampling point^42^.

In addition, the power spectrum ratio *Q* is defined as a parameter to measure the strength of the wrist muscle state. Under different training modes, the *Q* value is different, and the isometric and isotonic motion of the muscle can be recognized. The equation for the calculation of the *Q* value is given below.

19 $$Q=\frac{{F}_{0}}{F}=\frac{{\int }_{g_{0}-\delta f}^{g_{0}+\delta f}{F}_{g}dp}{{\int }_{-\infty }^{+\infty }{F}_{g}dp}$$

where *F* is the power spectrum, *F*_0_ is the power spectrum component at the peak, *g*_0_ is the frequency corresponding to the maximum power spectrum, and δ*f* is the unit frequency increment value.

The foregoing two methods need to only amplify, rectify, and filter the EMG signal, which are easy to calculate and can be widely used in feature extraction of EMG signals. For illustration, the AAV, VAR, and *Q* results are shown in Table 2. It is observed that in the rehabilitation modes of abduction–adduction (no. 1–3), flexion–extension (no. 4–6), and supination-pronation (no. 7–9), the corresponding *Q* values are different. The *Q* value does not change under the same rehabilitation training mode. Essentially, *Q* indicates the peak area of the power spectrum and the total area occupied by the power spectrum. Since the energy of the EMG signal is concentrated at the peak value, regardless of the maximum value of the subject’s EMG signal at the frequency, its characteristic value *Q* will remain stable. This characteristic is very meaningful for general applications because different people produce different frequencies at which the power spectrum peaks appear, while the trend of the variation in the surface EMG signal during muscle movement is stable. Thus, the *Q* value can be adopted for evaluation in general.

**Table 2:** AAV, VAR, and Q results for three rehabilitation modes.

Number	AAV	VAR	Q
1	9.703	1.924	0.402
2	10.105	2.055	0.402
3	9.455	1.897	0.402
4	6.442	10.025	0.186
5	7.051	11.255	0.186
6	6.035	10.004	0.186
7	12.205	4.223	0.704
8	13.116	5.021	0.704
9	12.824	4.658	0.704

Depending on the degree of wrist injury, different modes of rehabilitation training can be selected. The system implements multiple sets of training programs with LabVIEW software, which makes the rehabilitation robot more versatile and improves the patient’s autonomy in training mode selection.

## Discussion and conclusion

In this work, a new soft parallel robot dedicated to automated wrist rehabilitation tasks was designed. The robot employs an improved 6-SPS/PS parallel mechanism structure, which is driven by six PAM actuators and a linear electric cylinder. An EMG sensor is adopted to provide feedback for the rehabilitation robot system. Experimental studies verify that the developed robot meets the expected requirements. It is able to drive the patient’s wrist to achieve abduction–adduction, flexion–extension, and supination-pronation motions. In addition, different training programs, such as minor rehabilitation training and extreme rehabilitation training, have been developed and stored, allowing patients to choose suitable training modes based on their specific injuries. Such flexibility improves the versatility and autonomy of rehabilitation training. Compared with previous work, the developed soft wrist rehabilitation robot exhibits prominent superiority in terms of safety, adaptability, flexibility, cost, convenient operation, etc. The compact and lightweight structure of this robot makes it easy to use at home or in a hospital. In future work, more evaluations will be conducted to assess the rehabilitation training process. An EMG sensor-based feedback control system will be implemented, and a human–robot remote interaction will be designed to make the wrist rehabilitation robot more intelligent and convenient for practical use.

Soft materials are becoming increasingly widely used in robots due to their high safety and flexibility. As a representative example of such materials, PAM actuators are made of rubber material, which can be expanded and contracted by applying air pressure. A PAM control scheme is easy to realize. The designed rehabilitation robot is driven by six PAM actuators. This robot exhibits a high degree of flexibility, adaptability, safety, and mobility, is easily operated, and has a compact footprint. To demonstrate the superiority of this soft wrist rehabilitation robot, its performance is qualitatively compared with those of typical wrist rehabilitation robots described in the literature. The results are shown in Table 3, which reveals the superiority of the proposed design over previous work.

**Table 3:** Performance comparison with previous wrist rehabilitation robots.

Property	DOF	Structure	Security	Cost	Control
Ref^8^	2	Rigid	Low	High	Complicated
Ref^10^	2	Rigid	Low	High	Complicated
Ref^12^	3	Rigid	High	Low	Complicated
Ref^15^	3	Rigid	Low	Low	Simple
This work	3	Soft	High	Low	Simple

With the improved mechanical design of this work, the required training motions are achieved in a given space. This approach overcomes the disadvantages of traditional rehabilitation equipment, which has bulky, expensive, and heavy structures with limited mobility. Notably, open-loop control is implemented in the present work, which is sufficient for realizing the required wrist rehabilitation motion. In future work, closed-loop control will be realized to achieve more accurate control results^43^, and human–machine interface technology will be introduced to realize remote control of the mechanism based on the electroencephalogram (EEG) signal^44^. In addition, the adaptation feedback mechanism will be combined with clinical rehabilitation treatment to achieve a better wrist rehabilitation effect. Further development of relevant sensing, virtual reality, and navigation technologies will continue to promote the application of wrist rehabilitation robots.

## Method

The novel wrist rehabilitation soft robot is fabricated by adopting six PAMs (DMSP series, Festo AG & Co. KG)^45^ and one central electric cylinder. One end of the PAM is fixed at the base, and another end is connected to the movable platform by a short elastic steel wire (diameter: 0.7 mm). The central electric cylinder along with an upper passive spherical joint is introduced as the support and constraint limb (seventh limb) with a linear drive. The PAM actuator does not require a piston rod and has a shrinkable diaphragm. It exhibits fine dynamic performance, and the shrinkage length can reach up to 25% of the rated length. Moreover, it provides sufficient load, which is highly suitable as a soft actuator for wrist rehabilitation robots. The PAMs are controlled by using a pneumatic controller (EX600, SMC Corp.). As a linear actuator, the electric cylinder provides a trust force of 200 N and no-load speed of 45 mm/s, with a stroke of 100 mm and diameter of 20 mm.

To detect the healing effect for the wrist, a myoelectric sensor (EMG Sensor SX230, Biometrics Ltd.) was used to monitor the activity of the wrist and upper limb muscles^46^. The sensor module integrates a filtering and amplifying circuit to amplify the weak myoelectric signal (in the range of ± 1.5 mV) of the human body. The output signal is provided in analogue form with the range of 0–3.0 V, and the reference voltage is 1.5 V. The signals from surface-bonded EMG sensors are acquired with a sampling frequency of 2 kHz during the wrist training process. The EMG signal is obtained using a single-board microcontroller (Arduino MEGA2560, Arduino AG) with a serial port connection. The obtained analogue voltage signal is then transmitted to a personal computer running LabVIEW software for the Arduino toolkit.
